# Mathematical modeling predicts that endemics by generalist insects are eradicated if nearly all plants produce constitutive defense

**DOI:** 10.1038/s41598-024-74771-0

**Published:** 2024-10-28

**Authors:** Suman Chakraborty, Shalu Dwivedi, Stefan Schuster

**Affiliations:** 1 Department of Bioinformatics, Matthias Schleiden Institute, Friedrich Schiller University Jena, Ernst-Abbe-Pl. 2, Jena, 07743 Thuringia Germany; 2 International Max Planck Research School ‘Chemical Communication in Ecological Systems’, Jena, 07745 Thuringia Germany

**Keywords:** Susceptible plants, Constitutive defense, Mathematical modeling, Generalist insects, Endemic, Non-endemic, Stability analysis, Plant-herbivore interactions, Ecological modelling, Evolutionary ecology, Applied mathematics, Ecology, Evolution, Plant sciences

## Abstract

Plants with constitutive defense chemicals exist widely in nature. The phenomenon is backed by abundant data from plant chemical ecology. Sufficient data are also available to conclude that plant defenses act as deterrent and repellent to attacking herbivores, particularly deleterious generalist insects. In the wild, generalist species are usually not endemic, meaning they are not restricted to certain plant species in a region. Therefore, our objective is to inspect theoretically whether evolution of chemical defenses in all plant species eradicate an endemic by any generalist species. The objective is addressed by developing deterministic ordinary differential equations under the following conditions: Plants without constitutive defenses are susceptible to oviposition by generalist insects, while they become defended against generalists by storing chemical defenses. From the models, we explicitly obtain that a generalist-free stable state is only possible if the vast majority of all plant individuals have chemical defenses. The model also allows one to predict the highest possible percentage of undefended plant individuals, which may be considered as free-riders.

## Introduction

**Fig. 1:**
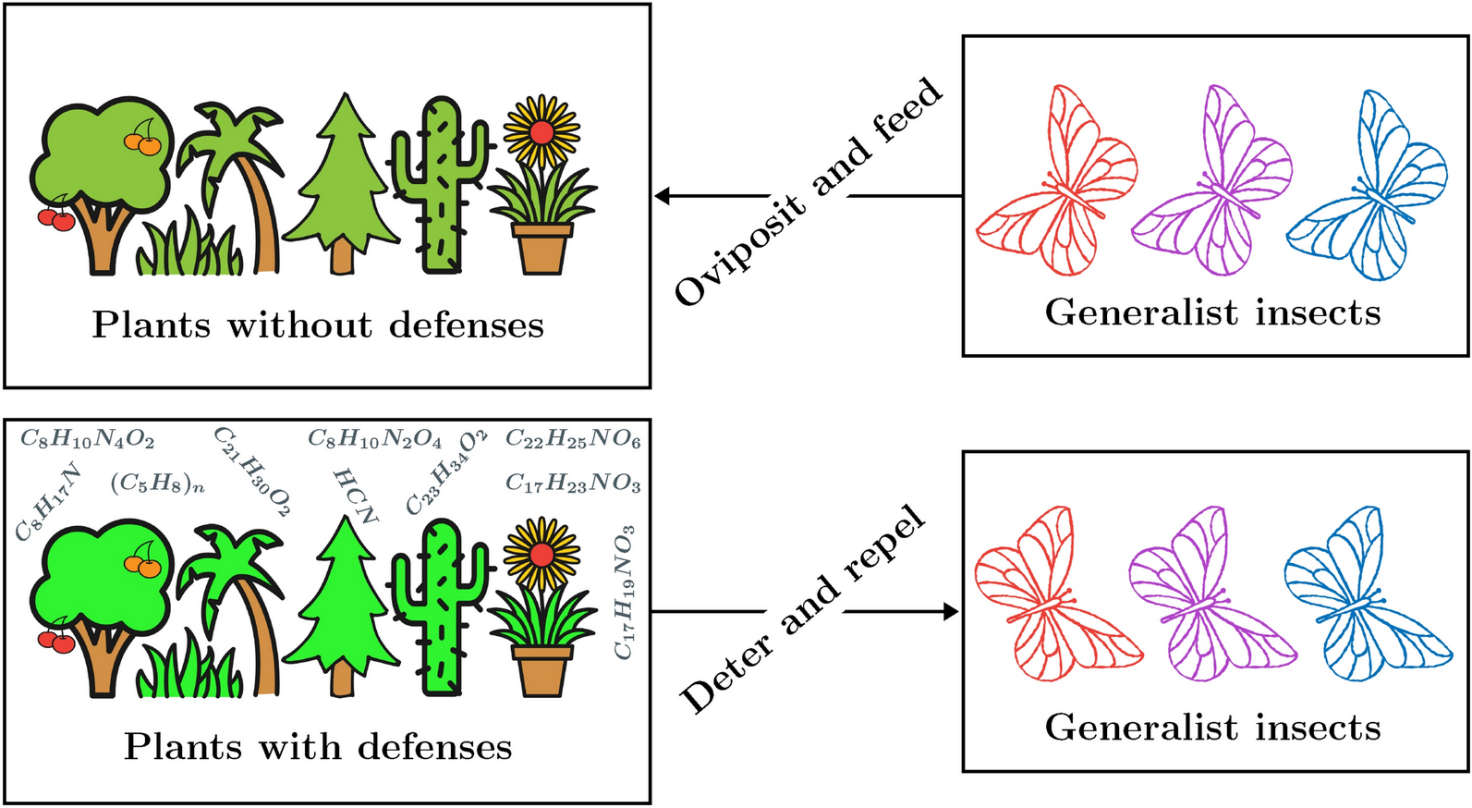
Plant defense vs generalist insects. Pictures drawn in “AutoDraw” (Google Creative Lab).

Plants store chemical defenses constitutively during their normal course of development^1–3^. Ample evidences of such defenses are available in nature^2,3^, as reviewed in the subsection ‘Examples of plant defenses’ below. Crucial roles of these constitutive defense compounds are to deter and repel deleterious generalist insects^4–8^. Several experimental studies tested the effects of plant defense. There is a slight difference in the meaning of plant deterrent and repellent chemicals. A plant deterrent is a chemical that prevents feeding and oviposition by insects, while a plant repellent is a chemical that causes insects to move away from its source^5,9,10^. If plants do not evolve constitutive defenses, then they are susceptible to generalist insects for oviposition. The phenomenon is expressed by Fig. 1.

According to some estimations, more than 100,000 different secondary compounds are found in plants^11–13^. In chemical ecology, a plausible theory could be that all individuals of every plant species have constitutive chemical defenses, even plants edible by humans^3,5,11,14^. Edible plants do involve some defense chemicals, which can, however, be tolerated due to their low concentrations and are often appreciated as flavors, like in black and white mustard or the numerous varieties of cabbage.

Although the theory that all plants have chemical defenses is confirmed by plentiful experimental evidence^3^, it has not been mathematically assessed yet. So, in this study, a proof of this theory is sought by building deterministic models, based on autonomous ordinary differential equations (ODEs). Specifically, the research question is whether any generalist insect species can become endemic if nearly all plant individuals (of all species) store chemical defenses. To be noted, generalist insects have a high diet breadth, but they do not feed permanently on specific plant species of a particular region^15–18^, which makes them non-endemic in the wild. This ability of being non-endemic or using multiple resources could have ecological and evolutionary advantage to generalists. Previous studies explained that generalists increase their fitness through access to various host plant species^18–21^. Although models have been proposed to understand the role of volatile and non-volatile organic compounds in plant-herbivore interactions^22–25^, it is not exactly clear how the non-endemic behavior of generalists is correlated to the multifaceted (ecological) roles of plant toxins.

**Fig. 2:**
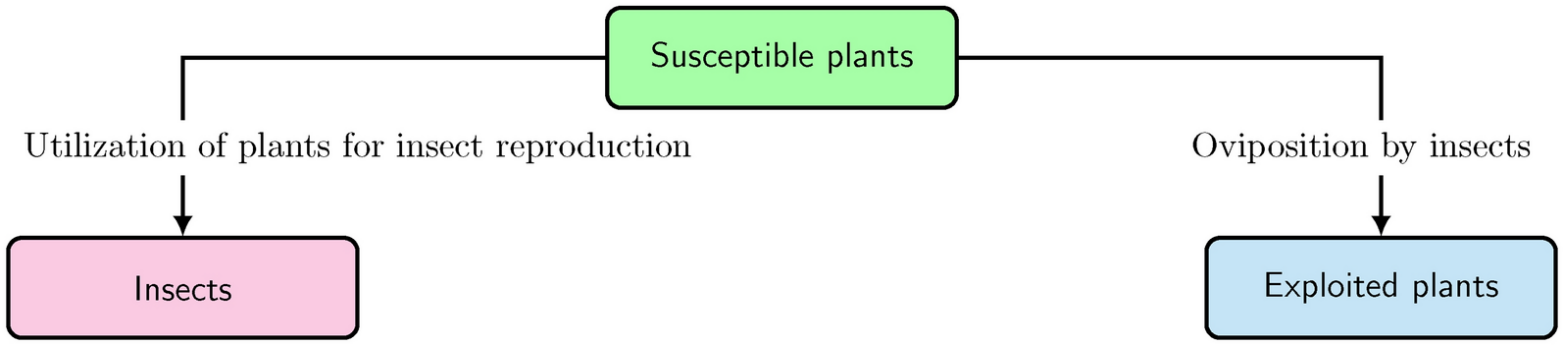
Basic scheme of the susceptible-exploited-insects model.

The main contributions of this study are two models and conclusions derived from them:

1. In the first model, a hypothetical situation is considered, where plants do not have constitutive defense compounds. This is done to investigate whether plants can avoid a generalist endemic without evolving chemical defenses. As depicted in Fig. 1, generalists oviposit on plants if defense compounds are absent. The undefended plants (of any species) are distinguished into two sections: Susceptible plants and exploited plants. Susceptible plants are likely to be exploited through oviposition by generalist insects, whereas exploited plants already have insect eggs (singly or in clutches) laid on them. The growth of the insect population is directly determined by the mean number of laid eggs. The flux across the compartments is expressed by the flowchart shown in Fig. 2. This model is influenced by the classic SIR model in epidemiology^26–29^.
2. The second model is based on the first one and includes, in addition, the phenomenon that new plants can germinate with chemical defenses and, thus, become defended against generalists. Defended plants are not susceptible to generalists, as expressed in Fig. 1. To formulate this model, the technique used for SIR models with vaccination (SIRV) or immunization^27–30^ is applied, where the total plant population includes the susceptible, exploited and defended plants. In case of plants, immunization could be the process of evolving chemical defenses to become defended against generalist herbivores^13,31^. From this model, one can investigate what percentage of plants have to germinate with chemical defenses to avoid a generalist endemic. That would theoretically explain the widespread occurrence of constitutive defense in plants^32–36^.

The paper structure is as follows: Models are built in two subsections of the “Models” section. Thereafter, the results of the models and future perspectives are expressed in the Discussion. Finally, the Conclusion is devoted to the main message of this study, model limitations and possible application domains. The required calculations for the stability analysis of the models are given separately in the Supplementary Information.

### Examples of plant defenses

Some examples of plants with defenses are provided by proanthocyanidins (PAs) in bilberry, peanuts, plums, cranberries, curry, and cinnamon plants^37^; flavonoids in fruits, vegetables, tea, cocoa and wine^38^; glucosinolates in the Brassicaceae plant family (including cabbage, rape and black mustard)^39,40^; caffeine in coffee, tea, cacao and kola plants^41,42^; nicotine in tobacco^43^; cannabinoids in cannabis^44^; terpenes and formylated phloroglucinol compounds (FPCs) in rose gum (*Eucalyptus grandis*)^45^; morphine in opium poppy^46^; cardenolides in milkweed^47^; atropine in deadly nightsade^3^; mimosine in Persian silk (Mimosa) and river tamarind (Leucaena) trees^48,49^; coniine in hemlock^50^; hydrogen cyanide (*HCN*) in cyanogenic plants, such as almonds, cassava, bamboo etc.^51^; colchicine in naked ladies (*Colchicum autumnale*) plant^52^ and so on.

Some examples regarding the effects of plant defenses on insects are given in the following. Proanthocyanidins act as feeding deterrents to the gypsy moth (*Lymantria dispar*), brown-tail moth (*Euproctis chrysorrhoea*) and winter moth (*Operophtera brumata*)^53–55^; flavonoids deter African armyworm (*Spodoptera exempta*), fall armyworm (*Spodoptera frugiperda*) and African cotton leafworm *Spodoptera littoralis*^56,57^; isoflavonoids are deterrents to cotton bollworm (*Helicoverpa armigera*), African cotton leafworm and fall armyworm (*S. frugiperda*)^58^; glucosinolates are deterrent and repellent to cabbage moth (*Mamestra brassicae*), southern armyworm (*Spodoptera eridania*), cabbage looper (*Trichoplusia ni*) and green peach aphid (*Myzus persicae*)^40^; caffeine deters beet armyworms and cotton aphids^59,60^; nicotine, cannabinoids and morphine can be deterrents to many insect herbivores^43,61,62^, and terpenes in lavender are repellent to the clothes moth^63^. Lavender oil is commercially used by humans to protect clothes from this insect^63^. Furthermore, cardenolides deter *Nephila* spiders^64^; atropine is a feeding deterrent to gypsy moth larvae, *Lymantria dispar*^65^; mimosine can suppress insect growth^66,67^; coniine and hydrogen cyanide (*HCN*) are poisonous to several groups of insects^68,69^; colchicine sterilizes *Bactrocera tau* (walker) fly and kills *Melanoplus differentialis* and *Gryllus assimilis*^70,71^ etc.

## Models

The deterministic dynamical system is based on three variables, $S,\, E$ and *I*. *S* is the number of susceptible plants, *E* is the number of exploited plants and *I* is the insect population at time *t*. The sum of susceptible and exploited plants is equal to the total plant population in the first model:

1 $$\begin{aligned} S + E = N \end{aligned}$$

where *N* denotes the total number of plants in a region. Moreover, initially, all plants are susceptible to generalist insects, so that the initial value of *S* reads:

2 $$\begin{aligned} S_0 = N \end{aligned}$$

Therefore, $E_0 = 0$ from Eq. (1). Insects can emerge (e.g. by invasion) in a group or singly initially, i.e. $I_0 > 0$.

### Model excluding plant defense

**Fig. 3:**
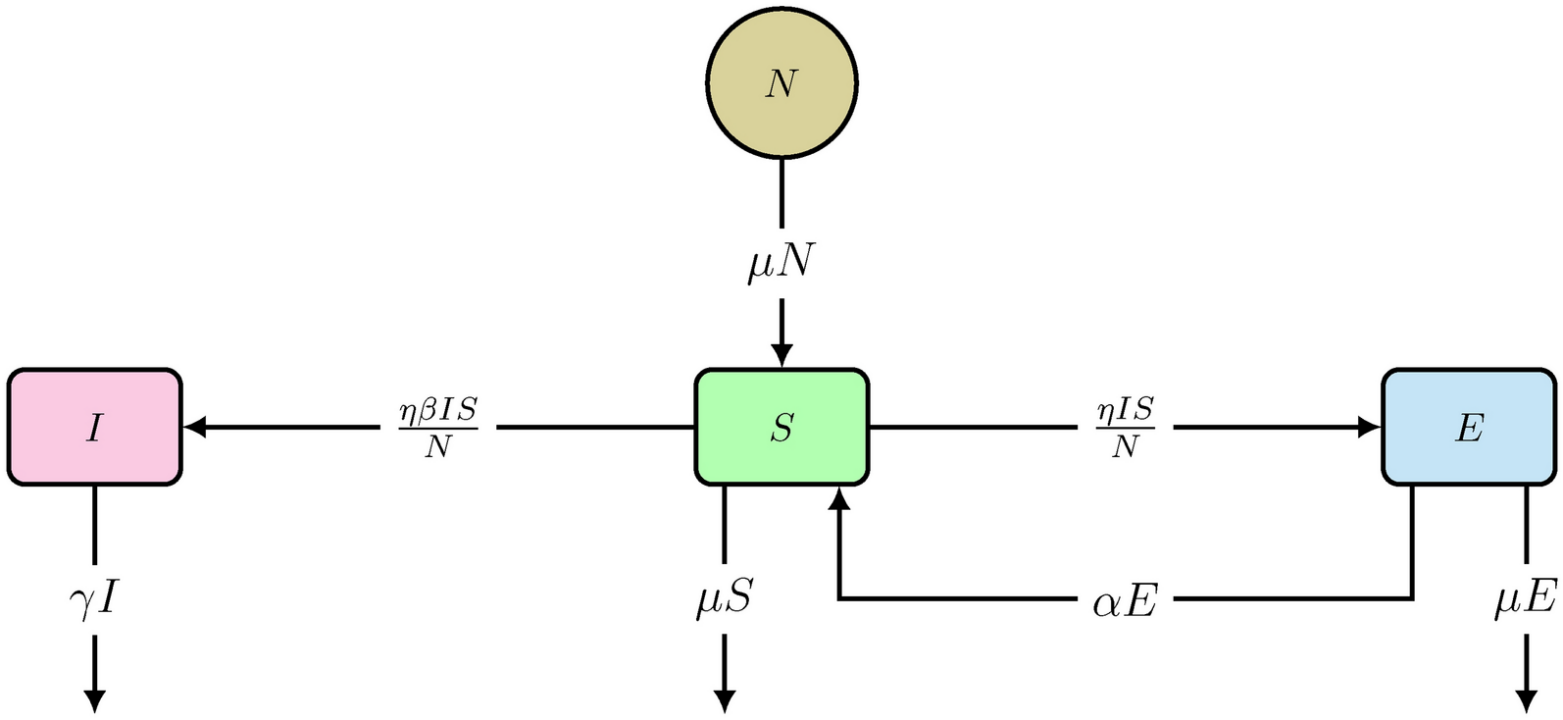
Scheme of the *SEI* model (3) with demographic effect and re-susceptibility in plants. Variables and parameters are explained in the text.

The first model is developed from the fluxes between the *S*, *E* and *I* compartments, including demography and re-susceptibility in plants, shown by the scheme in Fig. 3. So, it is named as *SEI* model. In that Figure, $\eta$ is the oviposition number (constant), i.e. the number of plants exploited by an insect per day, $\beta$ is the deposition number (constant), i.e. the mean number of eggs deposited by an insect per exploited plant per day and $\gamma$ is the natural death rate (constant) for an insect, which is the reciprocal of the average lifespan ($1/\gamma$) of an insect.

In order to keep $S + E = N$ constant, we assume that the germination (reproductive growth) and death rate constants (per capita) are the same in plants, denoted by $\mu$. Although somewhat artificial, this assumption is also made in many SIR type models to keep the total population constant^29,30^. Exploited plants can be re-susceptible to insects attack, because a plant can be exploited (by insects) multiple times in its lifetime. Let $\alpha$ be the per capita rate at which exploited plants become re-susceptible. The rate equations are:

3a $$\begin{aligned} \frac{dS}{dt}\,=\,\mu N - \eta I \frac{S}{N} - \mu S + \alpha E \end{aligned}$$

3b $$\begin{aligned} \frac{dE}{dt} = \eta I \frac{S}{N} - \mu E - \alpha E \end{aligned}$$

3c $$\begin{aligned} \frac{dI}{dt} = \beta \eta I \frac{S}{N} - \gamma I \end{aligned}$$

where the germination rate constant of plants is proportional to the total plant population (*N*) and all new germinated plants are susceptible as well. Since $S+E=N$ (Eq. (1)), the sum of the above Eqs. (3a) and (3b) gives:

4 $$\begin{aligned} \frac{dN}{dt} = 0 \end{aligned}$$

Equation (4) confirms that the total plant population is a constant if the rate constants of plant germination and death are equal. The normalized term *S*/*N* in Eqs. (3a), (3b) and (3c) represents the prevalence of susceptibility. From the commencement of the attack, the insect population increases in Eq. (3c) if:

$$\begin{aligned} \frac{dI}{dt} > 0 \end{aligned}$$

which is, under the condition $I \ne 0$, equivalent to:

5 $$\begin{aligned} S> \frac{N \gamma }{\eta \beta } \Rightarrow R_0 > \frac{N}{S}, \quad \text {where}\, R_0 = \frac{\eta \beta }{\gamma } \end{aligned}$$

Since $S \le N$ from Eqs. (1) and (2), Eq. (5) leads to:

6 $$\begin{aligned} R_0 > 1 \end{aligned}$$

where $R_0$ is the reproduction number of an insect, i.e. the number of viable eggs laid by an insect in its lifetime. Since an insect can lay between 100 and 2000 eggs (mean values) in its lifetime^72–75^, we obtain $R_0 \gg 1$. So, the insect population (*I*) grows initially from the commencement of attack.

The model (3) has a non-endemic equilibrium (oviposition and generalist free):

7 $$\begin{aligned} H^*_{Free} = (S^*,E^*,I^*) =\left( N, \, 0, \, 0 \right) \end{aligned}$$

$H^*_{Free}$ is asymptotically stable for $R_0 < 1$, shown in the Supplementary Information S1 and illustrated in Figs. 5 and 6A. However, since all insects lay an exceptionally high number of eggs^74^, $R_0 < 1$ is practically impossible when host plants (*N*) are abundant in a region. In contrast, $H^*_{Free}$ becomes an unstable equilibrium (a saddle point) for $R_0 > 1$, as illustrated in Fig. 6B and the Supplementary Information S1. The instability of $H^*_{Free}$ for $R_0 > 1$ explains that plants cannot eradicate the generalists. Moreover, the model (3) has an endemic equilibrium (including oviposition and generalist insects) for $R_0 > 1$:

8 $$\begin{aligned} H^*_{Endemic} = \left( \frac{N}{R_0}, \, \frac{N (R_0 - 1)}{R_0}, \, \frac{N (\mu + \alpha ) (R_0 - 1)}{\eta } \right) \end{aligned}$$

$H^*_{Endemic}$ is an asymptotically stable equilibrium, which is either a stable node or a stable focus, the proof is given in the Supplementary Information S2. It is important to note that all three variables are positive in the endemic state. This implies, in particular, that even in the long run, a certain percentage of plants is susceptible. The reason is that permanently, some plants are germinating (with birth rate constant $\mu$) and some are returning to the susceptible state (with rate constant $\alpha$). Persistence of the insect population creates a problem for plants, because then the insects continue damaging plants in the stable endemic state, shown by the time-courses in Fig. 7. Therefore, the conditions of model 3 are not sufficient to stop an endemic by generalists. However, generalists are usually non-endemic in the wild^19,76–79^, including generalist insect species^15–18^. So, an advancement is made in model (3) to verify whether evolution of constitutive defenses in plants can end the generalist endemic.

### Model including plant defense

**Fig. 4:**
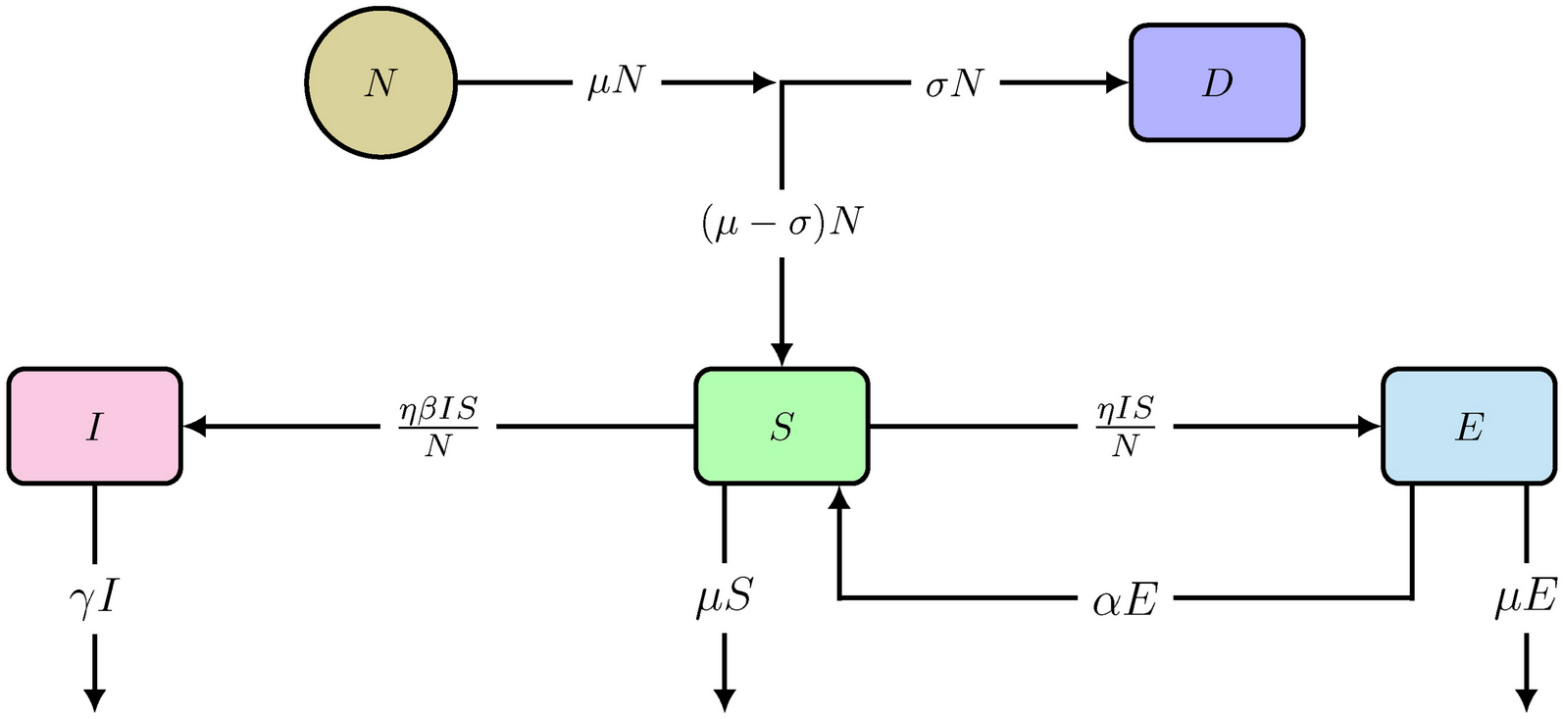
Scheme of the *SEDI* model (9) with demographic effect, re-susceptibility and germinated plants defended against generalists. Variables and parameters are explained in the text.

A new parameter $\sigma$ is introduced, representing the per capita rate at which new plants germinate with chemical defenses. These defended plants, denoted by the compartment *D*, are not hosts to generalists. Obviously, $\sigma \le \mu$ (with $\mu$ being the per capita reproduction or germination rate constant). The fluxes between the compartments are as shown in Fig. 3 with an additional flux to the compartment of defended plants (*D*) and a modification in the flux entering the susceptible compartment, expressed by the scheme in Fig. 4.

The rate equations are:

9a $$\begin{aligned} \frac{dS}{dt}\,=\,(\mu - \sigma ) N - \frac{\eta I S}{N} - \mu S + \alpha E \end{aligned}$$

9b $$\begin{aligned} \frac{dE}{dt} = \frac{\eta I S}{N} - (\mu + \alpha )\, E \end{aligned}$$

9c $$\begin{aligned} \frac{dD}{dt}\,=\, \sigma N - \mu D \end{aligned}$$

9d $$\begin{aligned} \frac{dI}{dt}\,=\, \frac{\beta \eta I S}{N} - \gamma I \end{aligned}$$

where the germinating plants enter the susceptible compartment at a per capita rate $(\mu - \sigma )$, shown in Fig. 4. The model (9) has an additional ODE representing the growth rate in defended plants (*D*), Eq. (9c). This main structural difference between models (3) and (9) allows us to assess the benefit of chemical defense evolved in plants. This new model (9) is called *SEDI* model, where the total plant population (*N*) is equal to the sum of susceptible, exploited and defended plants ($S+E+D = N$). Thus, the sum of Eqs. (9a), (9b) and (9c) gives:

10 $$\begin{aligned} \frac{dN}{dt} = 0 \end{aligned}$$

Eq. (10) proves that the total plant population is a constant in model (9) if the germination and death rate constants (per capita) are assumed to be equal.

The four-dimensional model (9) can be reduced to three dimensions, because *D* is the only variable occurring in the ODE (9c). The solution of that equation is not needed (although it can easily be found analytically) since the implicit functions of $S,\, E$ and *I* in the right-hand sides of the ODEs (9a), (9b) and (9d) are sufficient to analyze the behavior of model (9) in view of equilibria in $\mathbb {R}^3$ and their stability. The model (9) has two possible equilibrium points. The non-endemic (oviposition and insect free) equilibrium is:

11 $$\begin{aligned} Q^*_{Free} = \left( (1-p)N, \, 0, \, 0 \right) \end{aligned}$$

where $p = \frac{\sigma }{\mu }$ is the fraction of germinated plants (per capita) that evolved constitutive defense. To be noted $p \in [0,1]$, because $\sigma \le \mu$. Interestingly, some susceptible plants ($(1-p) N$) are not oviposited by generalist insects for $p < 1$. $Q^*_{Free}$ is asymptotically stable for $R_0 < 1$, proved in the Supplementary Information S3 and illustrated by Figs. 9 and 10B. For $R_0 > 1$, $Q^*_{Free}$ is asymptotically stable if and only if:

12 $$\begin{aligned} p > 1-\frac{1}{R_0} \end{aligned}$$

In the extreme case $p=0$, this is equivalent to the condition $R_0 < 1$ mentioned above. Calculations of deriving $Q^*_{Free}$ and the proof of its asymptotic stability for $R_0 > 1$ are given in the Supplementary Information S3. The time-course and phase-portrait of stable $Q^*_{Free}$ at $R_0 > 1$ are illustrated by Figs. 8 and 10A, respectively.

The endemic equilibrium of the model (9) is:

13 $$\begin{aligned} Q^*_{Endemic} = \left( \frac{ N}{R_0}, \, \left( 1 - p - \frac{1}{R_0} \right) N, \, \left( 1 - p - \frac{1}{R_0} \right) \frac{(\mu + \alpha ) N R_0}{\eta } \right) \end{aligned}$$

Generalists are non-endemic in the wild if the endemic equilibrium (13) does not exist at all, i.e. the insect and oviposition free equilibrium ($Q^*_{Free}$) is the only possible stable equilibrium. This is achieved if and only if the inequality (12) holds true, because exploited plants and generalists would be negative in the equilibrium (13) for $p > 1- 1/R_0$. In contrast, the endemic equilibrium of generalists exists and is stable (either a stable node or a stable focus) if $p < 1 - 1/R_0$, shown by the time-courses in Fig. 11. The derivation and stability analysis of $Q^*_{Endemic}$ are given in the Supplementary information S4. Although $Q^*_{Free}$ is stable for $R_0 < 1$ shown by Figs. 9 and 10B, the case is only feasible if the host plants of generalist insects are quite low in a region.

It is interesting to obtain that evolution of constitutive defense in plants depends on the reproduction number ($R_0$) of insects. Condition (12) can be interpreted as follows. In order to stop an endemic by generalist insects, not all plant individuals need to store chemical defenses. For example, if a generalist insect species has $R_0 = 500$, then $1 - 1/R_0 = 0.998$. Therefore, that particular generalist species cannot be endemic if more than $99.8 \%$ of all plants germinate with deterrent chemicals. Since $R_0 \gg 1$ for all insect species^74^, condition (12) also proves that if nearly all plants are germinated with chemical defenses, generalist insects become non-endemic in the wild. This phenomenon is shown by Figs. 8 and 10A. In other words, a few plants can remain undefended because if insects appeared (as a fluctuation of the free equilibrium), then these few plants would be insufficient to enable sufficient reproduction of insects.

**Fig. 5:**
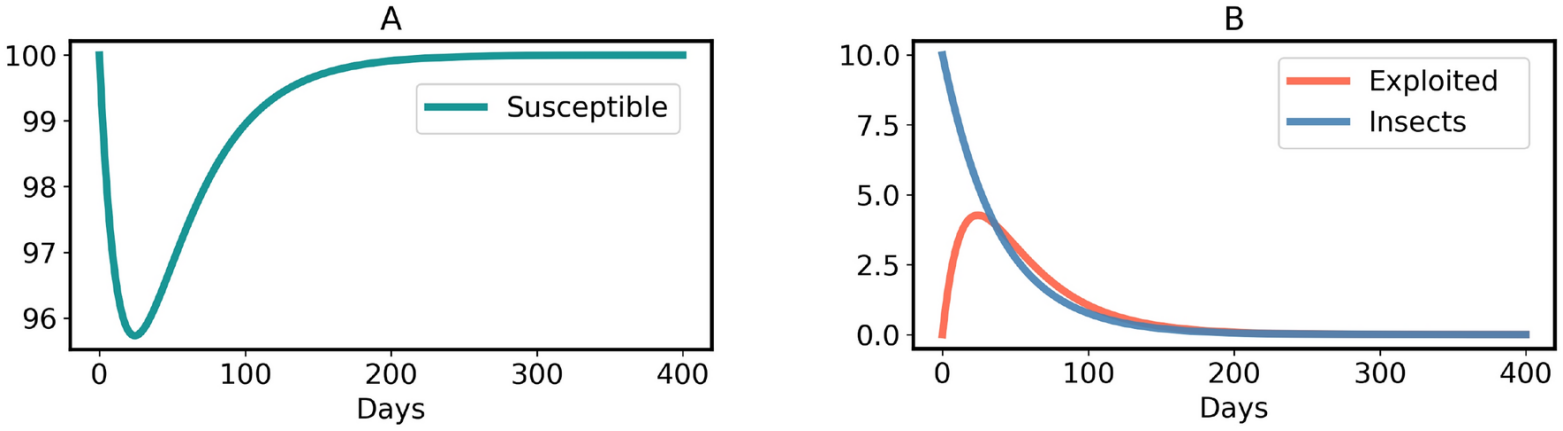
Time-courses (**A**, **B**) of model (3) converge to $H^*_{Free}$ when $R_0 < 1$. Parameters: $S_0 = 100, \eta = 0.05, \beta = 0.5, \gamma = 1/20, \mu = 0.05$ and $\alpha = 0.01$.

**Fig. 6:**
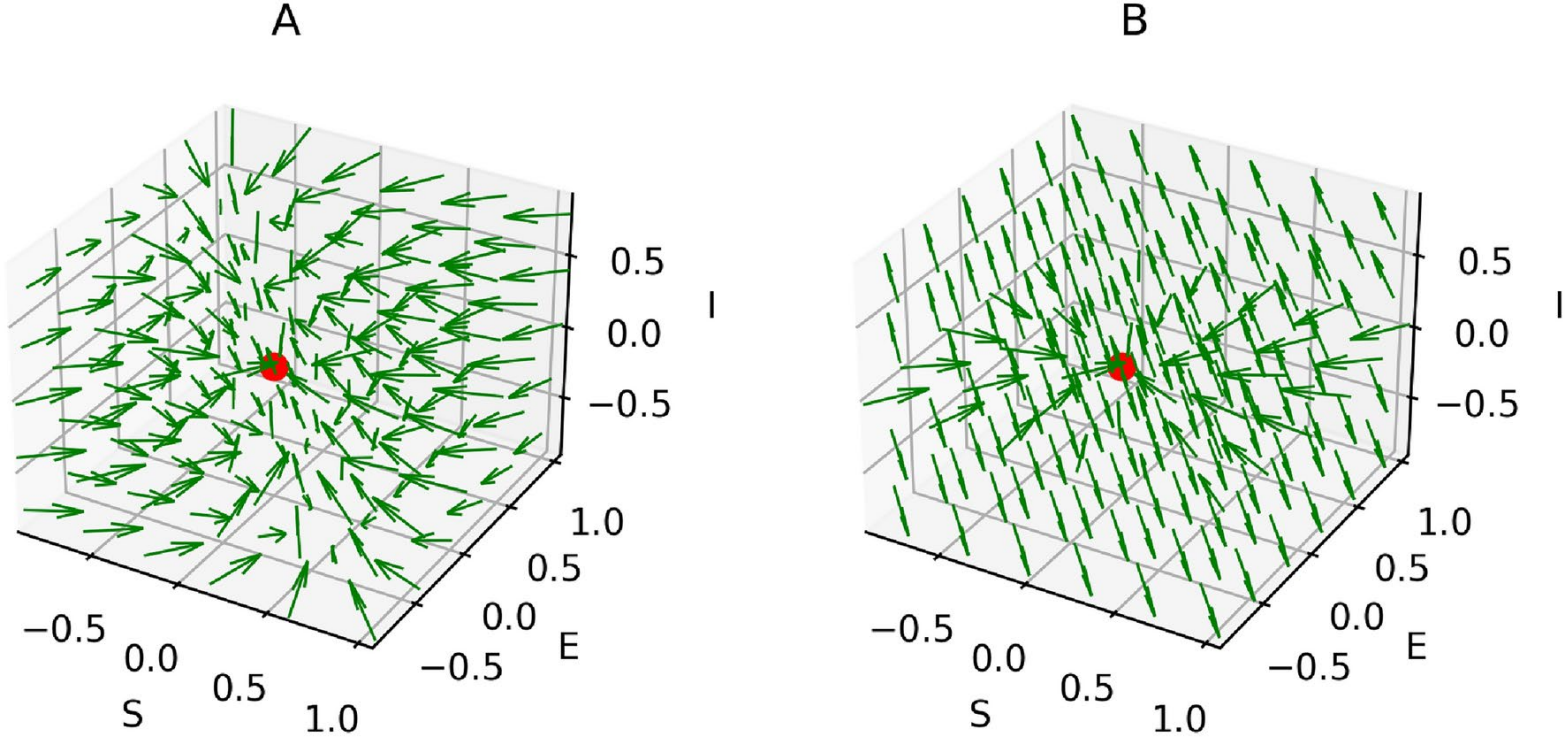
Phase portraits of $H^*_{Free}$, obtained from model (3). (**A**) Stable for $R_0 < 1$. Parameters are the same as in Fig. 5. (**B**) Unstable for $R_0 > 1$. Parameters: $\eta = 4, \beta = 0.5, \gamma = 1/20, \mu = 0.05$ and $\alpha = 0.01$.

**Fig. 7:**
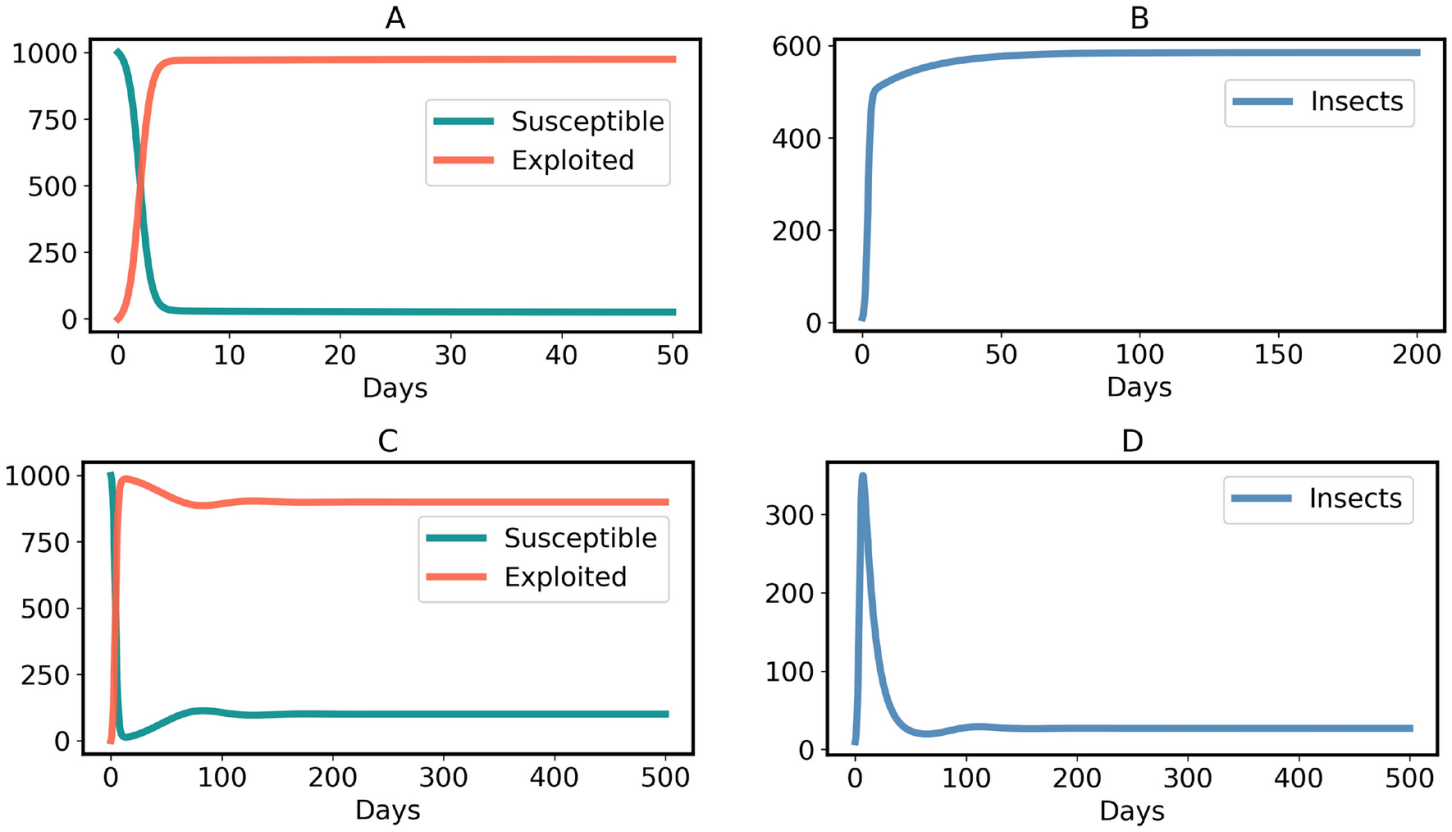
Time-course of model (3) when $R_0 > 1$. (**A**, **B**) Case where $H^*_{Endemic}$ is a stable node. Parameters: $\eta = 4, \beta = 0.5, \gamma = 1/20, \mu = 0.05$ and $\alpha = 0.01$. (**C**, **D**) Case where $H^*_{Endemic}$ is a stable focus. Parameters: $\eta = 2, \beta = 0.5, \gamma = 1/10, \mu = 0.005$ and $\alpha = 0.001$.

**Fig. 8:**
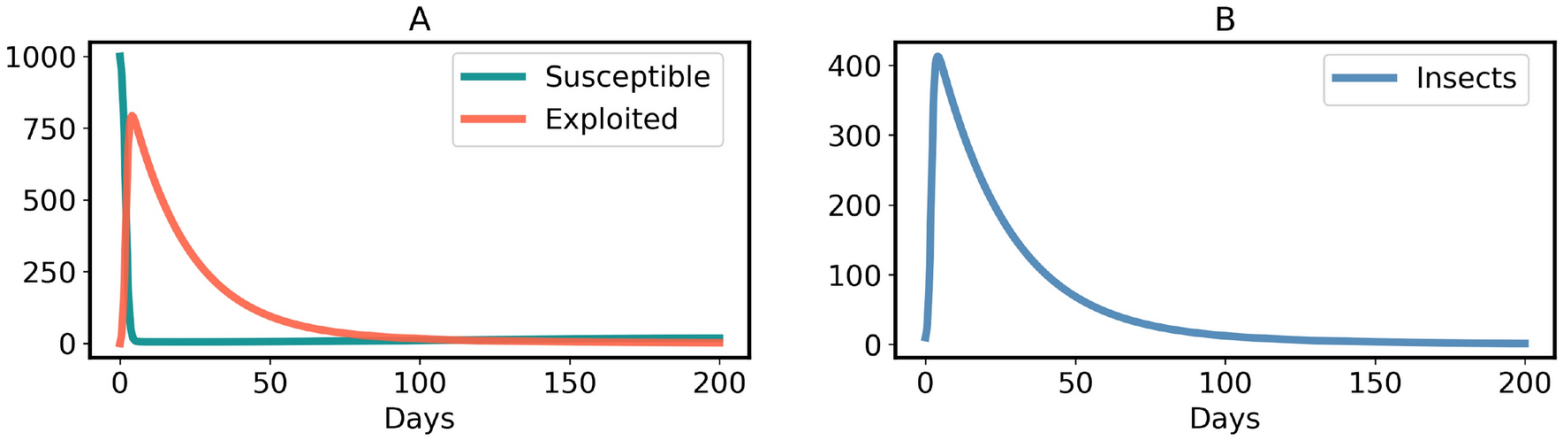
Time-courses of model (9) converge to $Q^*_{Free}$ when $p > 1-\frac{1}{R_0}$. Parameters: $\eta = 4, \beta = 0.5, \gamma = 1/20, \mu = 0.05, \sigma = 0.049$ and $\alpha = 0.01$.

**Fig. 9:**
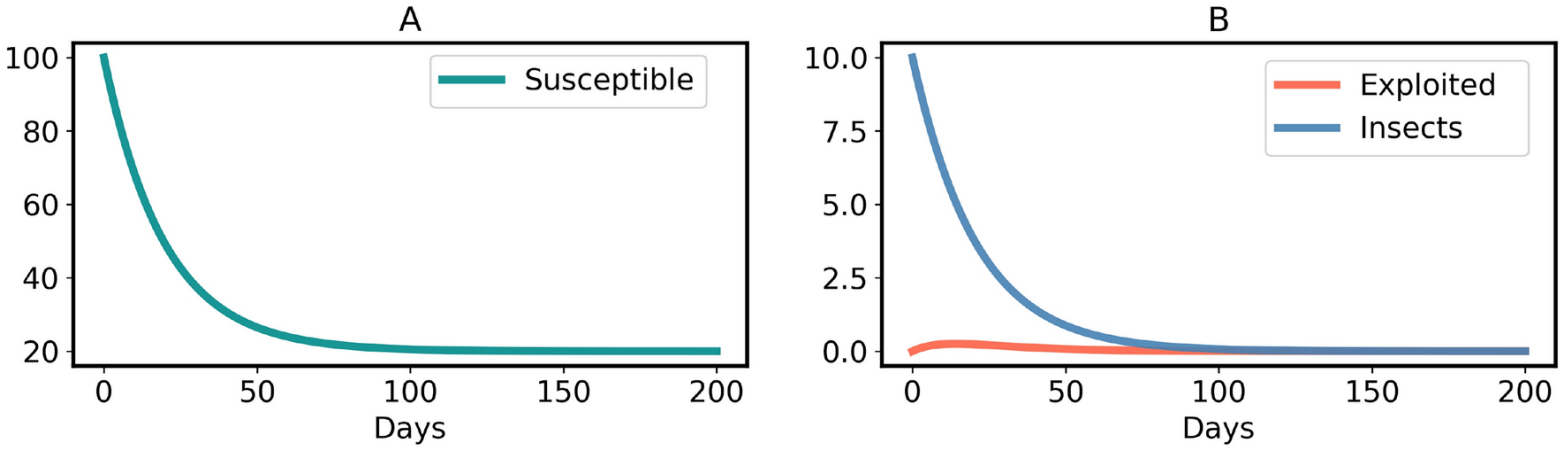
Time-courses (**A**, **B**) of model (9) converge to $Q^*_{Free}$ when $R_0 < 1$. Parameters are the same as in Fig. 8 except for $\eta = 0.05$.

**Fig. 10:**
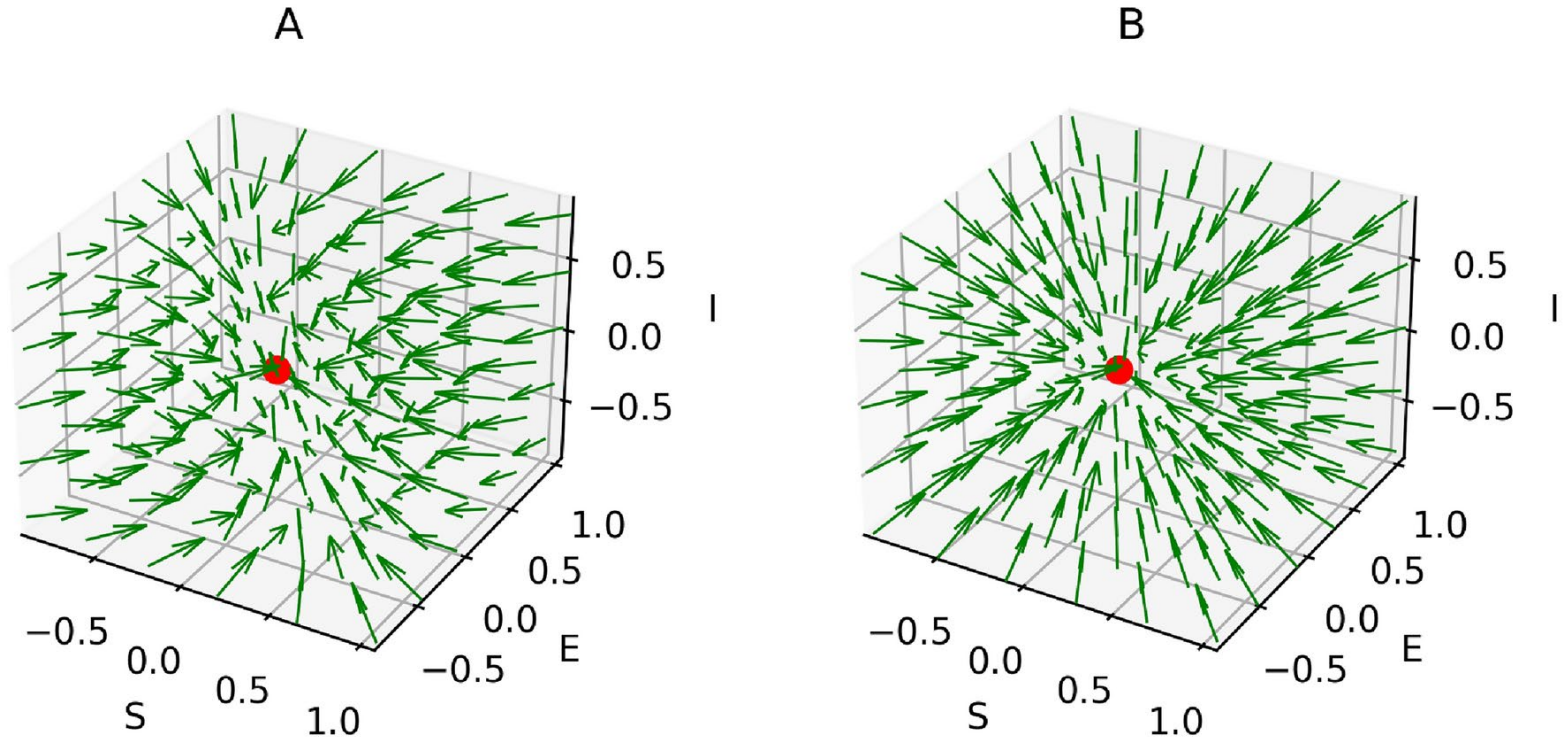
Phase portraits of $Q^*_{Free}$, obtained from model (9). (**A**) Stable for $R_0 > 1$ and $p > 1 - \frac{1}{R_0}$. Parameters: $\eta = 4, \beta = 0.5, \gamma = 1/20, p = \sigma /\mu = 0.99$ and $\alpha = 0.01$. (**B**) Stable for $R_0 < 1$. Parameters are the same as in Fig. 9.

**Fig. 11:**
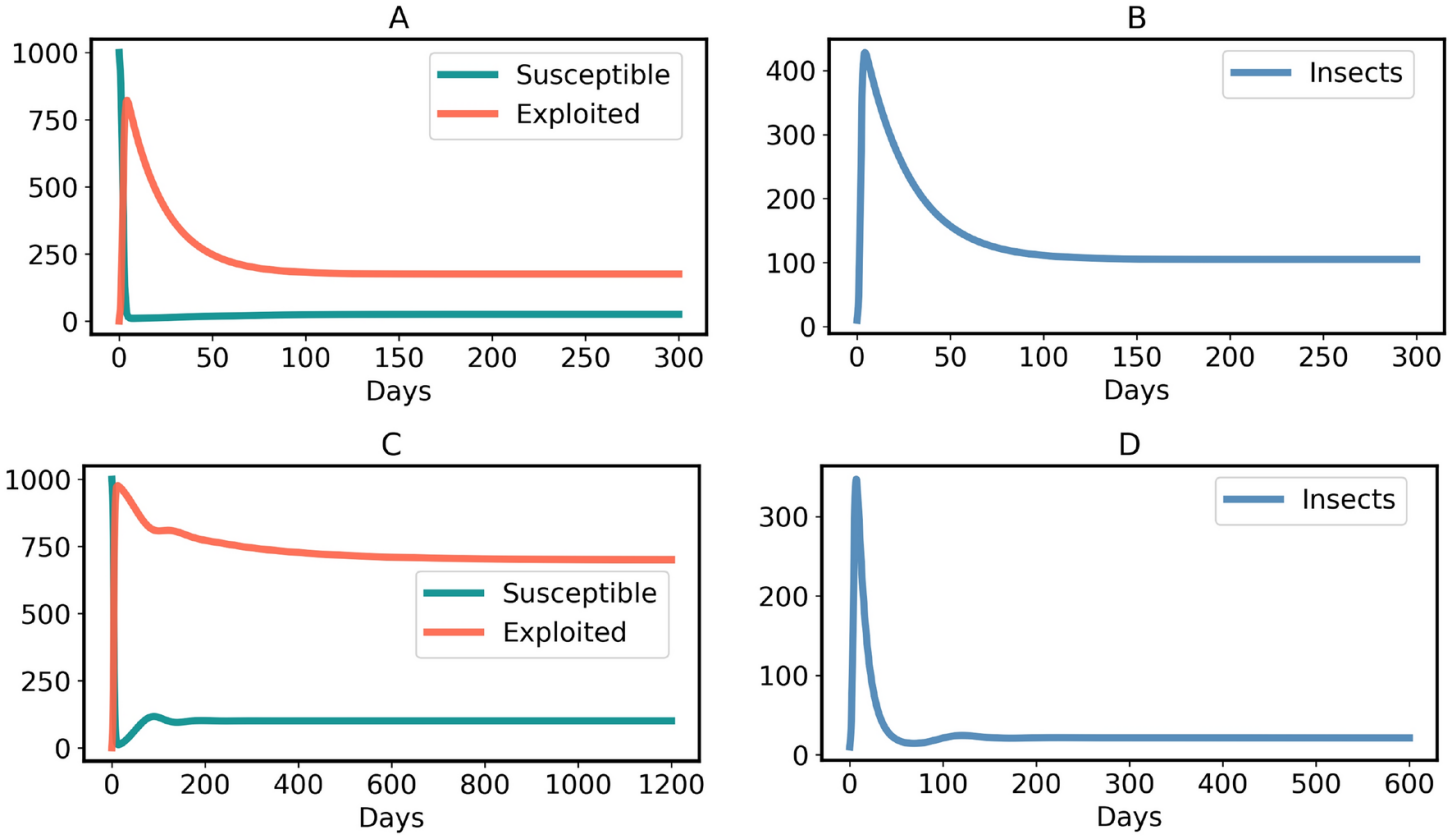
Time-course of model (9) for $p < 1-\frac{1}{R_0}$. (**A**, **B**) Case where $Q^*_{Endemic}$ is a stable node. Parameters: $\eta = 4, \beta = 0.5, \gamma = 1/20, \mu = 0.05, \sigma = 0.04$ and $\alpha = 0.01$. (**C**, **D**) Case where $Q^*_{Endemic}$ is a stable focus. $\eta = 2, \beta = 0.5, \gamma = 1/10, \mu = 0.005, \sigma = 0.001$ and $\alpha = 0.001$.

## Discussion

The main results of this study are as follows:

- If plants do not evolve chemical defenses, an endemic by generalist insects happens for $R_0 > 1$, Fig. 7. That result means generalists become native herbivores of plants in a certain region. However, as stated before, such phenomenon is not evident in the wild^15–18^.
- If a fraction of all plants are germinated with chemical defenses, but condition (12) is not fulfilled, the generalist endemic persists, Fig. 11. However, this result is not consistent with the observations mentioned in the previous point^15–18^.
- Plants display a generalist-free (non-endemic) stable equilibrium for $R_0 > 1$ if and only if nearly all plants are germinated with chemical defenses (i.e. constitutive plant defense), expressed by condition (12) and shown in Figs. 8 and 10A. That practically (though not literally) justifies the theory that all plants have chemical defenses^3,5,11,14^ due to the nonexistence of generalist endemic species in the wild^15–18^.
- If the reproduction number ($R_0$) of generalist insects in a region is below one, they cannot be endemic, Figs. 9 and 10B. This case is feasible when sufficient host plants are unavailable.

Condition (12) is necessary and sufficient for the generalist insect species to be non-endemic. That means $Q^*_{Free}$ is the only stable steady state if $p > 1 - 1/R_0$ and $p > 1 - 1/R_0$ occurs if $Q^*_{Free}$ is the only stable steady state, where $R_0 > 1$. Since we obtained that nearly all plants should germinate with chemical defenses, some exceptions could be possible in plant chemical ecology. These exceptions could be considered as free-riders. The concept of free-riders is often used in game theory^80,81^. However, the costs of producing defense chemicals are neglected for the sake of simplicity. Therefore, the free-riders do not have any physiological advantages in comparison to defended plants. If costs were included, as is done in many game-theoretical models^80,81^, a dilemma occurs. The free-riders then have a higher growth rate and could outcompete defended plants, so that the population becomes susceptible to herbivores. Various ways of resolving the dilemma have been proposed in biological contexts other than plant-herbivore interactions, such as spatial structure^82^.

Interestingly, a few plants can be found that may not invest sufficiently in constitutive defense^35,83,84^. For example, blue lupin (*Lupinus angustiflius*) may not produce any alkaloids even after suffering from herbivory^85^ and the invasive plant *Lespedeza cuneata* is inadequately defended constitutively against herbivory^86^. However, such examples are rare in plant chemical ecology. The basic model (3) without plant defense could be written as a model without vital dynamics, that is, without germination and death rates, and still having the conservation relation $S + E = N$. However, for the model (9) with plant defense, considering the plant germination rate is important to describe constitutive defense, which is present in plants during the normal course of development from germination on^1,2^.

The method is developed under the framework of stability theory. Stability theory is widely used in epidemiology^28–30,87^, eco-epidemiology^88–91^, population dynamics^27,92^ and other fields of mathematical biology or ecology^28,29^ to determine the asymptotic properties of solutions or equilibrium points in a long interval of time. In this field of study, the biological or ecological phenomena are expressed by a system of differential equations^27,92^. Stable equilibria of that system refer to the fixed points, where the entire system converge^27,29,92^. For example, vaccinating a certain percentage of a population eradicates an epidemic, because vaccination above the threshold makes the endemic-free equilibrium of the SIRV model (SIR model with vaccination) asymptotically stable^28,29^. Similarly, from model (9), it can be claimed that nearly all plants have evolved chemical defenses, because only then the generalist endemic free equilibrium is asymptotically stable.

Generalist insects are polyphagous by nature^93,94^, thus, select a wide range of host plants^16^. Nevertheless, plant defense negatively affects them due to their lack of specialism^95–98^. For example, glucosinolates and their hydrolyzed toxic isothiocyanate products of the Brassicaceae plant family reduce the development rate and cause high mortality of lepidopteran generalists^98,99^, such as *Spodoptera exigua, Spodoptera littoralis, Mamestra brassicae, Trichoplusia ni*, and *Helicoverpa armigera*^96^. Specialist insects, on the other hand, have evolved efficient counter-defense or resistance against host plant toxins^95,97,99^. Moreover, specialist insects use plant defense chemicals as a cue to identify host plants for oviposition^33,36,100–102^. Thus, plant defense could increase the pressure of specialist insect herbivores^33,36^. That is a direct contrast between the selection behavior of specialist vs generalist insects. It is not exactly clear how plants are benefiting against specialists by evolving defenses. However, plant defenses can attract the natural enemies of insect herbivores^36,103^, which leads to the tritrophic interaction^104^. That can be a possible explanation.

The indirect role of plant defense compounds and their subsequent products is attracting predators and parasitoids of deleterious insects^33,36,103–105^. For example, in *Pieris rapae* infested *Arabidopsis* plants, nitriles (a less toxic hydrolyzed products of glucosinolates) can recruit the parasitoid wasp *Cotesia rubecula*^106^; isothiocyanates in the Brassica plants can recruit *Trichogramma chilonis* wasps during infestation by *Plutella xylostella*^107^; entomopathogenic nematodes are recruited by insect-damaged maize roots^108^. Tritrophic interaction by herbivore-induced plant volatiles (HIPVs) has been modelled before^109,110^, where the emergence of natural enemies reduce the deleterious insect population. Although in this study, the indirect role of plant defenses is not considered, it could be an interesting refinement of the model (9).

A study conducted on microorganisms showed that the generalist genera are older than specialist genera in an evolutionary timescale^111^. Therefore, it is possible that the fundamental cause for plant species evolving chemical defenses was to eradicate the deleterious generalists^4–8^. However, for a holistic understanding, it is important to investigate the plants’ benefit of evolving chemical defenses against different group of deleterious insects (generalists and specialists) together^33,36^. Especially, it is known that specialists cause damage by being locally endemic to specific niches^111,112^. This leaves us with multiple questions: Whether tritrophic interaction is capable of ending a specialist-driven endemic, whether the chemical defense of plants is responsible for the endemics by specialists etc. It is worthwhile tackling these questions by mathematical modeling in the future.

## Conclusion

From the main results of this study, it can be concluded that generalist insects are non-endemic in the wild, because nearly all plants are germinated with secondary metabolites. However, that does not mean generalists become extinct. They can survive with a low reproduction number ($R_0 < 1$) on host plants. Although clear results are obtained, the main limitation of these models is the assumption of a constant total population of plants, i.e. $S+E = N$ in model (3) and $S+E+D = N$ in model (9), where *N* is constant. It would be intriguing to verify the results by advancing these models to describe overall growing plant populations^109,110^, in analogy to vital dynamics in SIR models^29,30^.

The study fits within the context of plant protection, insect behavior and pest control issues, which could be interesting not only for wildlife practitioners, but also for crop protection. Since generalists are only temporary visitors, crop protection measures should be more relevant against endemic specialist insect pests^36^. Several pest control methods, such as application of insecticides^113^, intercropping or mixed cropping^114^, sterile insect technique (SIT)^115^ etc. are practiced to save crop plants from deleterious insects. Including these control measures will be promising extensions to the model (9), which could expedite the eradication process of insect pests. Moreover, since plants are susceptible to both specialists and generalists, it would be significant to expand the model for both groups of insect (pest) species.

## Supplementary Information

Supplementary Information.

## Data Availability

All data generated or analyzed during this study are included in this published article [and its supplementary information file].
