# Supplementary material for: Mathematical modeling predicts that endemics by generalist insects are eradicated if nearly all plants produce constitutive defense
Source: Sci Rep. 2024 Oct 28;14:25771. doi: 10.1038/s41598-024-74771-0 (PMC11519633; doi:10.1038/s41598-024-74771-0)
Supplement: Supplementary file 1 — Supplementary Information. [file 41598_2024_74771_MOESM1_ESM.pdf]

Supplementary information for 'Endemics by generalist insects are eradicated if nearly all plants produce constitutive defense. An explanation by mathematical modeling'

Suman Chakraborty[1,2], Shalu Dwivedi[1], and Stefan Schuster[1,*]

[1]Dept. of Bioinformatics, Matthias Schleiden Institute, Friedrich Schiller University Jena, Ernst-Abbe-Pl. 2, 07743 Jena, Germany
[2]International Max Planck Research School 'Chemical Communication in Ecological Systems', 07745 Jena, Germany
[*]Correspondence: stefan.schu@uni-jena.de

June 13, 2024

This supplementary information contains the derivation and stability analysis of the equilibrium points: H^*_{Free}, $H^*_{Endemic}$, Q^*_{Free} and $Q^*_{Endemic}$, respectively.

S1. Derivation and stability of H^*_{Free}

The equilibrium H^*_{Free} is obtained from the equations:

$$\frac{dS}{dt} = \mu N - \frac{\eta IS}{N} - \mu S + \alpha E = 0 \tag{1a}$$

$$\frac{dE}{dt} = \frac{\eta IS}{N} - (\mu + \alpha) E = 0 \tag{1b}$$

$$\frac{dI}{dt} = \frac{\beta\eta IS}{N} - \gamma I = 0 \tag{1c}$$

From eq. (1c), we obtain:

$$\left(\frac{\beta\eta S}{N} - \gamma\right) I = 0 \tag{2}$$

Eq. (2) has two solutions:

- **Case 1:** $I = 0$.
- **Case 2:** If $I \neq 0$, then $S = \gamma N/\eta\beta = N/R_0$, where $R_0 = \eta\beta/\gamma$.

Here we analyze only case 1. Case 2 is analyzed in **S2**. Using $I = 0$ in eq. (1b), we obtain $E = 0$. Using $I = 0$ and $E = 0$ in eq. (1a), we obtain:

$$S = N$$

Therefore, the non-endemic equilibrium is: $H^*_{Free} = (N, 0, 0)$.

The Jacobian matrix from system 1 is:

$$M = \begin{pmatrix} -\frac{\eta I}{N} - \mu & \alpha & -\frac{\eta S}{N} \\ \frac{\eta I}{N} & -(\mu + \alpha) & \frac{\eta S}{N} \\ \frac{\beta \eta I}{N} & 0 & \frac{\beta \eta S}{N} - \gamma \end{pmatrix}$$

At H^*_{Free} the Jacobian is:

$$M_{H^*_{Free}} = \begin{pmatrix} -\mu & \alpha & -\eta \\ 0 & -(\mu + \alpha) & \eta \\ 0 & 0 & \beta\eta - \gamma \end{pmatrix}$$

The characteristic equation of the Jacobian is:

$$\begin{vmatrix} -\mu - \lambda & \alpha & -\eta \\ 0 & -(\mu + \alpha) - \lambda & \eta \\ 0 & 0 & \beta\eta - \gamma - \lambda \end{vmatrix} = 0$$

$$\Rightarrow (-\mu - \lambda)(-(\mu + \alpha) - \lambda)(\beta\eta - \gamma - \lambda) = 0$$

$$\Rightarrow \lambda = -\mu,\ -(\mu + \alpha),\ (\beta\eta - \gamma)$$

H^*_{Free} is asymptotically stable if all the eigenvalues (λ) are negative real numbers. Therefore, if $\beta\eta - \gamma < 0$ or $R_0 < 1$ (where $R_0 = \beta\eta/\gamma$), then H^*_{Free} is a stable node. In contrast, if $\beta\eta - \gamma > 0$ or $R_0 > 1$, then one of the eigenvalues is positive. Thus, H^*_{Free} becomes a saddle point, which is unstable.

S2. Derivation and stability of $H^*_{Endemic}$

The equilibrium $H^*_{Endemic}$ is also obtained from the differential equations given in **S1**.

Here, we analyze the case 2 mentioned in **S1**, where $S = N/R_0$. Using this value of S in eqs. (1b) and (1a), we get:

$$\frac{\eta IS}{N} - (\mu + \alpha)\,E = 0 \Rightarrow E = \frac{\eta I}{R_0(\mu + \alpha)}$$

$$\begin{aligned}
\frac{dS}{dt} = \mu N - \frac{\eta IS}{N} - \mu S + \alpha E &= 0 \\
\Rightarrow \mu N - \frac{\eta I}{R_0} - \frac{\mu N}{R_0} + \frac{\alpha \eta I}{R_0(\mu + \alpha)} &= 0 \\
\Rightarrow \left(\frac{\alpha\eta}{R_0(\mu+\alpha)} - \frac{\eta}{R_0}\right) I &= -\mu N \left(1 - \frac{1}{R_0}\right) \\
\Rightarrow \left(\frac{\alpha\eta - \mu\eta - \alpha\eta}{R_0(\mu+\alpha)}\right) I &= -\frac{\mu N(R_0 - 1)}{R_0} \\
\Rightarrow \left(\frac{\mu\eta}{(\mu+\alpha)}\right) I &= \mu N(R_0 - 1) \\
\Rightarrow I &= \frac{N(\mu+\alpha)(R_0-1)}{\eta}
\end{aligned}$$

Using this value of I at $E = \eta I/R_0(\mu + \alpha)$, we get:

$$E = \frac{N(R_0 - 1)}{R_0}$$

Hence, the endemic equilibrium is:

$$H^*_{Endemic} = \left(\frac{N}{R_0}, \frac{N(R_0-1)}{R_0}, \frac{N(\mu+\alpha)(R_0-1)}{\eta}\right)$$

The Jacobian matrix from system 1 is:

$$M = \begin{pmatrix} -\frac{\eta I}{N} - \mu & \alpha & -\frac{\eta S}{N} \\ \frac{\eta I}{N} & -(\mu+\alpha) & \frac{\eta S}{N} \\ \frac{\beta\eta I}{N} & 0 & \frac{\beta\eta S}{N} - \gamma \end{pmatrix}$$

At the endemic equilibrium $H^*_{Endemic}$, the Jacobian is:

$$M_{H^*_{Endemic}} = \begin{pmatrix} -(\mu+\alpha)(R_0-1) - \mu & \alpha & -\frac{\eta}{R_0} \\ (\mu+\alpha)(R_0-1) & -(\mu+\alpha) & \frac{\eta}{R_0} \\ \beta(\mu+\alpha)(R_0-1) & 0 & \frac{\beta\eta}{R_0} - \gamma \end{pmatrix}$$

The characteristic equation of the Jacobian is:

$$\begin{vmatrix} -(\mu+\alpha)(R_0-1)-\mu-\lambda & \alpha & -\frac{\eta}{R_0} \\ (\mu+\alpha)(R_0-1) & -(\mu+\alpha)-\lambda & \frac{\eta}{R_0} \\ \beta(\mu+\alpha)(R_0-1) & 0 & \frac{\beta\eta}{R_0}-\gamma-\lambda \end{vmatrix} = 0$$

$$\Rightarrow ((\mu+\alpha)(R_0-1)+\mu+\lambda)((\mu+\alpha)+\lambda)(\frac{\beta\eta}{R_0}-\gamma-\lambda)-\alpha(((\mu+\alpha)(R_0-1))(\frac{\beta\eta}{R_0}$$

$$-\gamma-\lambda)-\frac{\beta\eta}{R_0}(\mu+\alpha)(R_0-1))-\frac{\eta}{R_0}((\mu+\alpha+\lambda)(\beta(\mu+\alpha)(R_0-1)))=0$$

$$\Rightarrow -\lambda(\lambda+\mu+\alpha)(\lambda+\mu+(\mu+\alpha)(R_0-1))+\alpha(\lambda+\frac{\eta\beta}{R_0})(\mu+\alpha)(R_0-1)$$

$$-\frac{\eta\beta}{R_0}(\lambda+\mu+\alpha)(\mu+\alpha)(R_0-1)=0, \quad \text{where } \frac{\beta\eta}{R_0}=\gamma$$

$$-\lambda(\lambda+\mu)(\lambda+\mu+\alpha)-\lambda(\lambda+\mu+\alpha)(\mu+\alpha)(R_0-1))+(\mu+\alpha)(R_0-1)(\alpha\lambda-(\lambda+\mu)\frac{\eta\beta}{R_0})=0$$

$$-\lambda(\lambda+\mu)(\lambda+\mu+\alpha)-(\mu+\alpha)(R_0-1)(\lambda+\mu)(\lambda+\frac{\eta\beta}{R_0})=0$$

$$-\lambda^2(\lambda+\mu)-\lambda(\lambda+\mu)(\mu+\alpha)-(\mu+\alpha)(R_0-1)(\lambda+\mu)(\lambda+\frac{\eta\beta}{R_0})=0$$

$$(\lambda+\mu)(\lambda^2+(\mu+\alpha)R_0\lambda+\eta\beta(\mu+\alpha)(1-\frac{1}{R_0}))=0$$

$$\lambda=-\mu, -\frac{(\mu+\alpha)R_0}{2}\pm\frac{\sqrt{(\mu+\alpha)^2R_0^2-4\eta\beta(\mu+\alpha)(1-\frac{1}{R_0})}}{2}$$

The last two eigenvalues determine the stability of $H^*_{Endemic}$, because the first eigenvalue $(-\mu)$ is always a negative real number. Since $R_0 > 1$ or $1-1/R_0 > 0$, $\sqrt{(\mu+\alpha)^2R_0^2-4\eta\beta(\mu+\alpha)(1-1/R_0)} < (\mu+\alpha)R_0$ if the term inside the square root is a positive real number. If the term inside the square root is imaginary, the eigenvalues are complex conjugate numbers.

$H^*_{Endemic}$ is a stable node if all the eigenvalues are negative real numbers. Hence:

$$\begin{aligned} (\mu+\alpha)^2R_0^2 &\geq 4\eta\beta(\mu+\alpha)\left(1-\frac{1}{R_0}\right) \\ (\mu+\alpha)R_0 &\geq 4\gamma\left(1-\frac{1}{R_0}\right) > 0, \quad \text{since } \beta\eta=\gamma R_0 \end{aligned}$$

$H^*_{Endemic}$ is a stable focus if two of the eigenvalues are complex conjugate numbers with a negative real part. Hence:

$$(\mu+\alpha)R_0 < 4\gamma\left(1-\frac{1}{R_0}\right)$$

S3. Derivation and stability of Q^*_{Free}

The equilibrium Q^*_{Free} is obtained from the equations:

$$\frac{dS}{dt} = (\mu - \sigma)N - \frac{\eta IS}{N} - \mu S + \alpha E = 0 \tag{3a}$$

$$\frac{dE}{dt} = \frac{\eta IS}{N} - (\mu + \alpha)E = 0 \tag{3b}$$

$$\frac{dI}{dt} = \frac{\beta\eta IS}{N} - \gamma I = 0 \tag{3c}$$

From eq. (3c), we obtain:

$$\left(\frac{\beta\eta S}{N} - \gamma\right) I = 0 \tag{4}$$

Similar to the eq. (2) in **S1**, eq. (4) also has exactly two solutions:

- **Case 1:** $I = 0$.
- **Case 2:** The other solution is $S = N/R_0$, where $R_0 = \eta\beta/\gamma$.

We only analyze case 1 here. Case 2 is analyzed in **S4**. Using $I = 0$ at eq. (3b), we get $E = 0$. Using $I = 0$ and $E = 0$ in eq. (3a), we obtain:

$$\begin{aligned} S &= \frac{(\mu - \sigma)N}{\mu} \\ \Rightarrow S &= (1-p)N, \quad \text{where } \frac{\sigma}{\mu} = p \end{aligned}$$

Therefore, the non-endemic equilibrium is $Q^*_{Free} = ((1-p)N, 0, 0)$.

The Jacobian matrix from system 3 is:

$$M = \begin{pmatrix} -\frac{\eta I}{N} - \mu & \alpha & -\frac{\eta S}{N} \\ \frac{\eta I}{N} & -(\mu+\alpha) & \frac{\eta S}{N} \\ \frac{\beta\eta I}{N} & 0 & \frac{\beta\eta S}{N} - \gamma \end{pmatrix}$$

At the generalist free equilibrium Q^*_{Free}, the Jacobian is:

$$M_{Q^*_{Free}} = \begin{pmatrix} -\mu & \alpha & -\eta(1-p) \\ 0 & -(\mu+\alpha) & \eta(1-p) \\ 0 & 0 & \beta\eta(1-p) - \gamma \end{pmatrix}$$

The characteristic equation is:

$$\begin{vmatrix} -\mu - \lambda & \alpha & -\eta(1-p) \\ 0 & -(\mu+\alpha) - \lambda & \eta(1-p) \\ 0 & 0 & \beta\eta(1-p) - \gamma - \lambda \end{vmatrix} = 0$$

The eigenvalues are:

$$\lambda = -\mu,\ -(\mu+\alpha),\ \beta\eta(1-p)-\gamma$$

Q^*_{Free} is a stable node if all the eigenvalues are real and negative. Hence:

$$\begin{aligned} \beta\eta(1-p)-\gamma &< 0 \\ \Rightarrow R_0(1-p)-1 &< 0, \quad \text{since } R_0 = \frac{\beta\eta}{\gamma} \end{aligned}$$

The above inequality is obvious for $R_0 < 1$. The specific condition for the stability of Q^*_{Free} at $R_0 > 1$ is discussed below:

$$\begin{aligned} R_0(1-p)-1 &< 0 \\ \Rightarrow 1-p &< \frac{1}{R_0} \\ \Rightarrow p &> 1-\frac{1}{R_0} \end{aligned}$$

That is the stability condition of Q^*_{Free} for $R_0 > 1$. In contrast, if $p < 1 - 1/R_0$, then one of the eigenvalues $(\beta\eta(1-p)-\gamma)$ is positive. That makes Q^*_{Free} a saddle point, which is unstable.

S4. Derivation and stability of $Q^*_{Endemic}$

We also obtain the equilibrium $Q^*_{Endemic}$ from the differential equations given in **S3**.

Using the value $S = N/R_0$, mentioned in case 2 of **S3**, in eqs. (3b) and (3a), we obtain:

$$\frac{dE}{dt} = \frac{\eta I S}{N} - (\mu+\alpha)\,E = 0 \Rightarrow E = \frac{\eta I}{R_0(\mu+\alpha)}$$

$$\begin{aligned}
\frac{dS}{dt} = (\mu-\sigma)N - \frac{\eta I S}{N} - \mu S + \alpha E &= 0\\
\Rightarrow (\mu-\sigma)N - \frac{\eta I}{R_0} - \frac{\mu N}{R_0} + \frac{\alpha\eta I}{R_0(\mu+\alpha)} &= 0\\
\Rightarrow \left(\frac{\alpha\eta}{R_0(\mu+\alpha)} - \frac{\eta}{R_0}\right) I &= -\mu N\left(1-\frac{1}{R_0}\right) + \sigma N\\
\Rightarrow \left(\frac{\alpha\eta - \mu\eta - \alpha\eta}{R_0(\mu+\alpha)}\right) I &= -\frac{\mu N(R_0-1)}{R_0} + \sigma N\\
\Rightarrow \left(\frac{\mu\eta}{\mu+\alpha}\right) I &= \mu N(R_0-1) - \sigma N R_0\\
\Rightarrow \left(\frac{\eta}{\mu+\alpha}\right) I &= N(R_0-1) - pNR_0, \quad \text{where } p = \sigma/\mu\\
\Rightarrow I &= \frac{(\mu+\alpha)N(R_0-1-pR_0)}{\eta}\\
\Rightarrow I &= \left(1-p-\frac{1}{R_0}\right)\frac{(\mu+\alpha)NR_0}{\eta}
\end{aligned}$$

Using this value of I at $E = \eta I/R_0(\mu+\alpha)$, we get:

$$E = \left(1-p-\frac{1}{R_0}\right)N$$

Hence, the endemic equilibrium is:

$$Q^*_{Endemic} = \left(\frac{N}{R_0}, \left(1-p-\frac{1}{R_0}\right)N, \left(1-p-\frac{1}{R_0}\right)\frac{(\mu+\alpha)NR_0}{\eta}\right)$$

The Jacobian matrix from system 3 is:

$$M = \begin{pmatrix} -\frac{\eta I}{N} - \mu & \alpha & -\frac{\eta S}{N}\\ \frac{\eta I}{N} & -(\mu+\alpha) & \frac{\eta S}{N}\\ \frac{\beta\eta I}{N} & 0 & \frac{\beta\eta S}{N} - \gamma \end{pmatrix}$$

At the endemic equilibrium $Q^*_{Endemic}$, the Jacobian is:

$$M_{Q^*_{Endemic}} = \begin{pmatrix} -(\mu+\alpha)(R_0-pR_0-1) - \mu & \alpha & -\frac{\eta}{R_0}\\ (\mu+\alpha)(R_0-pR_0-1) & -(\mu+\alpha) & \frac{\eta}{R_0}\\ \beta(\mu+\alpha)(R_0-pR_0-1) & 0 & \frac{\beta\eta}{R_0} - \gamma \end{pmatrix}$$

The characteristic equation of the Jacobian is:

$$\begin{vmatrix} -(\mu+\alpha)(R_0-pR_0-1)-\mu-\lambda & \alpha & -\frac{\eta}{R_0} \\ (\mu+\alpha)(R_0-pR_0-1) & -(\mu+\alpha)-\lambda & \frac{\eta}{R_0} \\ \beta(\mu+\alpha)(R_0-pR_0-1) & 0 & \frac{\beta\eta}{R_0}-\gamma-\lambda \end{vmatrix} = 0$$

Replacing $\frac{\eta\beta}{R_0}$ by γ in the characteristic equation, we get:

$$\begin{vmatrix} -(\mu+\alpha)(R_0-pR_0 1)-\mu-\lambda & \alpha & -\frac{\eta}{R_0} \\ (\mu+\alpha)(R_0-pR_0 1) & -(\mu+\alpha)-\lambda & \frac{\eta}{R_0} \\ \beta(\mu+\alpha)(R_0-pR_0-1) & 0 & -\lambda \end{vmatrix} = 0$$

$\Rightarrow (\lambda+\mu+(\mu+\alpha)(R_0-pR_0-1))(\lambda+\mu+\alpha)(-\lambda)+\alpha(-\lambda(\mu+\alpha)(R_0-pR_0-1)-\frac{\eta R_0}{\beta}(\mu+\alpha)(R_0-pR_0-1))-\frac{\beta\eta}{R_0}(\lambda+\mu+\alpha)(\mu+\alpha)(R_0-pR_0-1)=0$

$\Rightarrow -\lambda(\lambda+\mu+(\mu+\alpha)(R_0-pR_0-1))(\lambda+\mu+\alpha)-\alpha(\mu+\alpha)(R_0-pR_0-1)(\lambda+\frac{\eta\beta}{R_0})-\frac{\eta}{R_0}(\beta(\lambda+\mu+\alpha)(\mu+\alpha)(R_0-pR_0-1))=0$

$\Rightarrow -\lambda(\lambda+\mu+(\mu+\alpha)(R_0-pR_0-1))(\lambda+\mu+\alpha)+(\mu+\alpha)(R_0-pR_0-1)(\alpha\lambda-(\lambda+\mu)\frac{\eta\beta}{R_0})=0$

$\Rightarrow -\lambda(\lambda+\mu)(\lambda+\mu+\alpha)-\lambda(\lambda+\mu+\alpha)(\mu+\alpha)(R_0-pR_0-1))+(\mu+\alpha)(R_0-pR_0-1)(\alpha\lambda-(\lambda+\mu)\frac{\eta\beta}{R_0})=0$

$\Rightarrow -\lambda(\lambda+\mu)(\lambda+\mu+\alpha)-(\mu+\alpha)(R_0-pR_0-1)(\lambda^2+\mu\lambda+(\lambda+\mu)\frac{\eta\beta}{R_0})=0$

$\Rightarrow -\lambda(\lambda+\mu)(\lambda+\mu+\alpha)-(\mu+\alpha)(R_0-pR_0-1)(\lambda+\mu)(\lambda+\frac{\eta\beta}{R_0})=0$

$\Rightarrow -\lambda^2(\lambda+\mu)-(\lambda+\mu)(\mu+\alpha)((R_0-pR_0-1)\frac{\eta\beta}{R_0}+\lambda(R_0-pR_0))=0$

$\Rightarrow (\lambda+\mu)(\lambda^2+\lambda(\mu+\alpha)(R_0-pR_0)+(\mu+\alpha)(R_0-pR_0-1)\frac{\eta\beta}{R_0})=0$

$$\Rightarrow \lambda=-\mu,-\frac{(\mu+\alpha)(1-p)R_0}{2}\pm\frac{\sqrt{(\mu+\alpha)^2(1-p)^2R_0^2-4(\mu+\alpha)(R_0(1-p)-1)\gamma}}{2}, \quad \text{since } \beta\eta=\gamma R_0$$

The first eigenvalue, $-\mu$, is a negative real number. Now we analyze the other two eigenvalues. Since $Q^*_{Endemic}$ exists when $p<1-1/R_0$, the term $(R_0(1-p)-1)$ inside the square root is positive. Therefore, $\sqrt{(\mu+\alpha)^2(1-p)^2R_0^2-4(\mu+\alpha)(R_0(1-p)-1)\gamma}$ is either a positive real number or an imaginary number. Moreover, the square root is less than $(\mu+\alpha)(1-p)R_0$ when it is a positive real number. Therefore, either all the eigenvalues are negative real numbers or two of them are complex numbers (one is the complex conjugate of the other).

$Q^*_{Endemic}$ is a stable node if all the eigenvalues are negative real numbers. Hence:

$$(\mu+\alpha)^2(1-p)^2R_0^2 \quad \geq \quad 4(\mu+\alpha)(R_0(1-p)-1)\gamma$$

$Q^*_{Endemic}$ is a stable focus if two of the eigenvalues are complex conjugate numbers with negative a real part. Hence:

$$(\mu+\alpha)(1-p)^2R_0^2<4(R_0(1-p)-1)\gamma$$
